# Maturation of arteriovenous fistulas in patients with and without preexisting hemodialysis catheters

**DOI:** 10.1016/j.amsu.2019.10.008

**Published:** 2019-10-11

**Authors:** Yuthapong Wongmahisorn

**Affiliations:** Department of Surgery, Faculty of Medicine Vajira Hospital, Navamindradhiraj University, 681 Samsen Road, Dusit District, Bangkok, 10300, Thailand

**Keywords:** Arteriovenous fistula, Catheter, Hemodialysis, Maturation

## Abstract

**Background:**

Central venous catheter (CVC) placement has been linked to systemic inflammation. This study was conducted to compare the successful maturation of arteriovenous fistulas (AVFs) and the preoperative white blood cell (WBC) profiles between patients with and without preexisting CVCs.

**Materials and methods:**

A retrospective cohort study was conducted with 550 patients who underwent first-time AVF creation. Patients were divided into three groups according to preexisting CVCs and CVC types as follows: tunneled CVC (*n* = 23), nontunneled CVC (*n* = 223), and no CVC (*n* = 304). These three groups were compared in terms of AVF maturation and preoperative WBC profiles.

**Results:**

The mean age of the patients was 61.1 ± 14.3 years. The AVF maturation rates of the tunneled CVC, nontunneled CVC and no CVC groups were 65.2%, 54.7% and 74.7%, respectively (*p* < 0.001). According to the uni- and multivariate analyses, only the nontunneled CVC group had a lower odds of AVF maturation compared to the no CVC group (adjusted odds ratio 0.43; 95% confidence interval 0.29–0.62). Patients with preexisting nontunneled CVC but not tunneled CVC also had significantly higher mean WBC and neutrophil counts but a lower percentage of lymphocytes than patients with no preexisting CVC.

**Conclusion:**

Preexisting nontunneled CVC had a negative impact on the successful maturation of the newly created AVF. Patients in the nontunneled CVC group had the highest preoperative WBC and neutrophil counts but the lowest lymphocyte percentage compared to patients in the other two groups.

## Highlights

• Nontunneled CVC has a negative impact on the successful maturation of AVF.
• AVF maturation could be affected by duration of CVC placement.
• Preexisting nontunneled CVC is significantly related to high preoperative WBC count.

## 1 Introduction

Hemodialysis is the most common method used to treat end-stage renal disease (ESRD) [1]. This procedure may be performed using a native arteriovenous fistula (AVF), arteriovenous graft, or central venous catheter (CVC) as a vascular access point [2]. Among the three access modalities, an AVF is considered the best option because of its longer patency and fewer complications [2]. Despite these advantages, the reported rates of hemodialysis initiation with an AVF among ESRD patients varied across countries, ranging from 14% to 66% [3,4].

In clinical practice, as many as 60%–80% of incident patients start their hemodialysis therapy with a CVC due to being unable to wait for the maturation of AVFs or having a condition in which AVF development is not feasible [3,5]. However, long-term catheterization carries an increased risk of various complications, including infection, central venous stenosis and thrombosis, which, in turn, decrease patient survival [6,7]. These complications occur particularly in nontunneled CVC users [[8], [9], [10]]. Hence, the recommendation is for a nontunneled CVC to be used for no more than three weeks [11]. Nevertheless, given financial issues and the patient preference for not removing a catheter unless necessary, nontunneled CVCs are commonly used for longer than three weeks [[8], [9], [10],[12], [13], [14]], especially in low-resource settings. Tunneled CVCs poses a lower risk of infection and can be used for longer durations [15]. However, they are more costly and require more skilled operators for their placement compared to nontunneled CVCs.

Among ESRD patients initiating hemodialysis with a CVC, the time at which they switch to an AVF varies in accordance with age, sex, medical condition and patient willingness [16,17]. Successful AVF maturation depends on various factors, including patient-related, preoperative, intraoperative and postoperative factors. Among these, a preexisting CVC has been shown to be associated with poor AVF maturation [18,19]. Recently, there have been many studies investigating the underlying mechanisms responsible for the occurrence of AVF failure. Several authors have noted the pivotal role of inflammation in neointimal hyperplasia, which is a foundation of AVF nonmaturation [[20], [21], [22]].

Previous studies have reported that CVC placement contributes to chronic inflammation independent of infection [23,24]. Nevertheless, no data exist to assess the impact of the inflammatory milieu on AVF maturation in relation to the presence or absence of preexisting dialysis catheters. The objective of this study was to compare the maturation success of newly created AVFs between patients who did not have preexisting CVCs and those who did. The preoperative inflammatory markers, as assessed by white blood cell (WBC) profiles, of patients with and without preexisting CVCs were also evaluated.

## 2 Materials and methods

### 2.1 Study design and eligibility criteria

This was a retrospective cohort study using electronic medical records of all consecutive patients with ESRD who were referred to the author's institution between January 2009 and December 2017 for AVF creation. The inclusion criteria were patients who received an AVF for the first time and had an available preoperative complete blood count (CBC) that had been measured in the institutional laboratory within a week before AVF surgery. Patients were divided into three groups according to whether they had a preexisting CVC and CVC type: patients with prior tunneled and nontunneled CVC placements were identified as the respective tunneled CVC and nontunneled CVC groups, whereas those without preexisting CVCs were labeled as the no CVC group. The exclusion criteria were as follows: 1) patients with hematologic disease, acute infectious disease, signs or symptoms of infection, or recent steroid intake (≤14 days); 2) patients who had their CVC insertion performed elsewhere; and 3) patients who were lost to follow-up. This study was approved by the Institutional Review Board (approval number 85/2561) and was performed in compliance with the Declaration of Helsinki.

### 2.2 Dialysis catheter insertion

The insertion of the tunneled CVC was performed by an experienced vascular surgeon, whereas the placement of the nontunneled CVC was performed by an attending nephrologist or vascular surgeon of the hospital. The insertion site of choice was the right internal jugular vein. If this venous site was not available for catheterization, the left internal jugular vein was then chosen, followed by the femoral vein, in that order.

### 2.3 Fistula creation and patient care

The type of primary AVF created could be radiocephalic or brachiocephalic based on the vascular surgeon's discretion in accordance with the feasibility of the available vessels. An end-to-side anastomosis was created between the cephalic vein and the radial or brachial artery, using continuous 6/0 or 7/0 polypropylene sutures under local anesthesia. No specific drug regimen was prescribed by a vascular surgeon post AVF creation. Patients who were prescribed antiplatelet agents or anticoagulants by nephrologists/cardiologists because of their medical diseases would be advised to continue their usual dose of medications in the postoperative period.

The preoperative WBC data were obtained from a CBC ordered by a nephrologist to check a patient's health status or from a CBC ordered by an attending surgeon for a preoperative evaluation of the patient. In the author's institution, an automated hematology analyzer model Unicel DxH 800 (Beckman Coulter, Inc., Brea, CA, USA) was used to measure all CBC specimens, including WBC count and WBC differential percentages. The machine was calibrated three times daily for quality control. The intra-assay coefficients of variation for WBC and differential counts were less than 3%, which fell within the acceptable range.

All patients were scheduled for follow-up visits at two weeks after AVF creation and then every month for a further 3- to 6-month period to assess AVF outcomes and complications. The first cannulation of the AVF was usually performed 6 weeks after the operation. If an AVF became unusable or any complications occurred, an additional surgical or endovascular intervention was applied to promote AVF patency.

### 2.4 Data collection and outcome definitions

Data for all included patients were extracted from the hospital electronic database. These included age, sex, body mass index (BMI), the presence or absence of preexisting dialysis catheters, the type and duration of CVC used (in patients with preexisting CVCs), comorbid conditions, current medications, preoperative WBC count and differential, and the presence or absence of AVF maturation.

The duration of CVC use was placed into one of three categories: <21 days, 21–89 days or ≥90 days. The author selected the cut-off duration of less than 21 days because it reflected the adherence to recommendations that a nontunneled CVC should be used for a period shorter than three weeks [11], whereas the cut-off duration of less than 90 days indicated a quality service of the renal unit in terms of the early referral of patients to vascular surgery specialists for AVF creation [15]. Preoperative WBCs were divided into two groups of high (above median) or low (below median) WBC levels. Comorbid conditions consisted of diabetes mellitus, hypertension, ischemic heart disease, cerebrovascular disease and cancer. Current medications included antithrombotic agents (antiplatelets or anticoagulants), statins, calcium channel blockers, angiotensin converting enzyme inhibitors/angiotensin II receptor blockers and beta blockers.

A diagnosis of AVF maturation in this study was based on functional maturation, which was defined as the successful use of the AVF for at least six consecutive dialysis sessions by the third month following its creation [25].

### 2.5 Statistical analysis

Data were analyzed using IBM SPSS Statistics version 22.0 (IBM Corporation, Armonk, NY, USA). Continuous data among the three patient groups are expressed as the means with standard deviations and were compared with one-way analysis of variance; when the overall analysis was significant, the intergroup comparisons were then made by the Scheffe method. Categorical variables are presented as numbers with percentages and were compared using the chi-square test. Using the no CVC group as the reference, the adjusted odds ratios with 95% confidence intervals (CIs) for AVF maturation in the tunneled CVC and nontunneled CVC groups were analyzed by multivariate logistic regression analysis adjusted for potential confounding factors. A value of *p* < 0.05 was considered statistically significant.

This study has been reported in line with the STROCSS criteria [26].

## 3 Results

In total, 550 patients were included in the study. Of these, 23 (4.2%) had preexisting tunneled CVCs, 223 (40.5%) had preexisting nontunneled CVCs, and 304 (55.3%) did not have prior CVC placement.

The patient characteristics at the time of first AVF creation are presented in Table 1. Their mean age was 61.1 ± 14.3 years (range 19–94 years); 277 (50.4%) were male, and 273 (49.6%) were female. There were no differences in age, sex, presence of comorbidities, and current medications among the three groups of patients. The mean BMI of patients with preexisting nontunneled CVCs but not of those with tunneled CVCs was significantly lower than that of patients with no preexisting CVC. The mean duration of CVC placement in patients with preexisting tunneled CVC was 175.5 ± 345.8 days, which was significantly longer than the duration of 29.5 ± 34.3 days in patients with preexisting nontunneled CVC (*p* < 0.001).

**Table 1:** Patient characteristics at the time of first arteriovenous fistula creation.

	Overall	Tunneled CVC group	Non-tunneled CVC group	No CVC group	*P* value
	(*n* = 550)	(*n* = 23)	(*n* = 223)	(*n* = 304)	
Age (years)	61.1 (14.3)	56.2 (17.5)	61.8 (13.7)	60.9 (14.5)	0.187^a^
Age group					0.741^b^
<65 years	312 (56.7)	16 (69.6)	123 (55.1)	173 (56.9)	
65–79 years	183 (33.3)	5 (21.7)	76 (34.1)	102 (33.6)	
≥80 years	55 (10.0)	2 (8.7)	24 (10.8)	29 (9.5)	
Sex					0.876^b^
Male	277 (50.4)	11 (47.8)	110 (49.3)	156 (51.3)	
Female	273 (49.6)	12 (52.2)	113 (50.7)	148 (48.7)	
Body mass index (kg/m^2^)	24.0 (4.5)	24.7 (5.7)	23.4 (4.6)*	24.5 (4.3)	0.021^a^
Comorbid conditions					
Diabetes mellitus	292 (53.0)	9 (39.1)	115 (51.6)	168 (55.3)	0.275^b^
Hypertension	472 (85.8)	18 (78.3)	190 (85.2)	264 (86.8)	0.494^b^
Ischemic heart disease	103 (18.7)	7 (30.4)	44 (19.7)	52 (17.1)	0.254^b^
Cerebrovascular disease	43 (7.8)	2 (8.7)	17 (7.6)	24 (7.9)	0.981^b^
Cancer	32 (5.8)	1 (4.3)	18 (8.1)	13 (4.3)	0.176^b^
Current medications					
Antithrombotic agents	227 (41.3)	8 (34.8)	92 (41.3)	127 (41.8)	0.806^b^
Statins	240 (43.6)	7 (30.4)	100 (44.8)	133 (43.8)	0.414^b^
Calcium channel blockers	363 (66.0)	11 (47.8)	142 (63.7)	210 (69.1)	0.074^b^
ACE inhibitors or ARBs	131 (23.8)	4 (17.4)	51 (22.9)	76 (25.0)	0.648^b^
Beta blockers	274 (49.8)	10 (43.5)	110 (49.3)	154 (50.7)	0.788^b^
Duration of CVC placement					<0.001^b^
<21 days	–	8 (34.8)	137 (61.4)	–	
21–89 days	–	7 (30.4)	68 (30.5)	–	
≥90 days	–	8 (34.8)	18 (8.1)	–	

Data are presented as the mean (standard deviation) or *n* (%).

ACE, angiotensin converting enzyme; ARB, angiotensin II receptor blocker; CVC, central venous catheter.

a One-way analysis of variance.

b Chi-square test. *p < 0.05 compared to the no CVC group.

Overall, the rate of AVF maturation was 66.2%. Table 2 compares AVF maturation among the three groups of patients. The maturation rate was lowest in the nontunneled CVC group, followed by the tunneled CVC and no CVC groups: 54.7%, 65.2% and 74.7%, respectively (*p* < 0.001). According to univariate analysis, only the nontunneled CVC group was associated with a lower odds of AVF maturation compared to the no CVC group. When multivariate analysis with adjustment for potential confounding factors, including age, sex and BMI, was performed, a preexisting nontunneled CVC retained its significance as an independent negative predictor of AVF maturation. The adjusted odds ratio was 0.43 (95% CI, 0.29–0.62). In other words, a history of prior nontunneled CVC placement was associated with a 2.4-fold (95% CI, 1.62–3.42) increase in the risk of AVF nonmaturation.

**Table 2:** Crude and adjusted odds ratios for arteriovenous fistula maturation among the three groups of patients.

	Tunneled CVC group	Non-tunneled CVC group	No CVC group^a^
	(*n* = 23)	(*n* = 223)	(*n* = 304)
AVF maturation, *n* (%)			
Yes (*n* = 364)	15/23 (65.2)	122/223 (54.7)	227/304 (74.7)
No (*n* = 186)	8/23 (34.8)	101/223 (45.3)	77/304 (25.3)
Crude OR (95% CI)	0.64 (0.26–1.56)	0.41 (0.28–0.59)	1.0
Adjusted OR^b^ (95% CI)	0.63 (0.26–1.55)	0.43 (0.29–0.62)	1.0

AVF, arteriovenous fistula; CI, confidence interval; CVC, central venous catheter; OR, odds ratio.

a Reference group.

b Adjusted for age, sex and body mass index.

Table 3 shows the preoperative WBC profiles in the three groups of patients. Compared to patients with no preexisting CVC, patients with preexisting nontunneled CVC but not those with tunneled CVC had significantly higher mean WBC and neutrophil counts but a lower percentage of lymphocytes. The percentages of neutrophils, eosinophils, monocytes and basophils and the numbers of lymphocytes, eosinophils, monocytes and basophils were not significantly different among the three groups of patients.

**Table 3:** Preoperative white blood cell and differential counts among the three groups of patients.

	Tunneled CVC group	Non-tunneled CVC group	No CVC group	*P* value^a^
	(*n* = 23)	(*n* = 223)	(*n* = 304)	
Total WBC count (/μL)	6782.2 (2209.5)	7352.0 (1890.3)*	6906.9 (1483.3)	0.008
Neutrophils				
Percentage	67.5 (12.6)	66.8 (10.0)	65.4 (8.7)	0.177
Number	4709.7 (2003.2)	4983.0 (1692.0)*	4549.1 (1280.4)	0.005
Lymphocytes				
Percentage	20.9 (10.3)	20.7 (8.0)*	22.6 (6.9)	0.019
Number	1304.3 (628.8)	1463.0 (562.9)	1532.7 (530.4)	0.084
Eosinophils				
Percentage	3.7 (3.4)	4.6 (3.8)	4.5 (3.4)	0.498
Number	244.6 (221.6)	340.5 (294.3)	311.5 (247.2)	0.177
Monocytes				
Percentage	7.5 (2.9)	7.0 (2.8)	7.0 (2.7)	0.727
Number	489.8 (205.5)	510.8 (229.4)	478.5 (200.1)	0.228
Basophils				
Percentage	0.4 (0.5)	0.5 (0.7)	0.5 (0.4)	0.966
Number	31.0 (36.0)	32.8 (48.1)	31.1 (31.9)	0.897

Data are presented as the mean (standard deviation). ^a^One-way analysis of variance test. *p < 0.05 compared to the no CVC group.

CVC, central venous catheter; WBC, white blood cell.

The effect of the duration of CVC placement on AVF maturation in relation to preoperative WBC levels was further explored among patients with preexisting CVCs, either tunneled or nontunneled types and is presented in Table 4. It appeared that the rate of AVF maturation was not affected by the duration of CVC placement if a patient had a low preoperative WBC level (below median or below 7200/μL) (*p* = 0.191). In contrast, in the event that a patient had a high preoperative WBC count (above median or above 7200/μL), the rate of AVF maturation was attenuated with increasing duration of CVC placement (*p* = 0.032).

**Table 4:** The effect of the duration of central venous catheter placement on arteriovenous fistulation maturation in relation to preoperative white blood cell levels among the three groups of patients.

	Arteriovenous fistula maturation, *n* (%)			
	Duration of CVC use			
	<21 days	21–89 days	≥90 days	*P* value
Low preoperative WBC level (<7200/μL)				
Tunneled CVC group (*n* = 11)	5/5 (100)	2/3 (66.7)	3/3 (100)	0.231
Non-tunneled CVC group (*n* = 109)	45/67 (67.2)	16/32 (50.0)	5/10 (50.0)	0.204
Both groups (*n* = 120)	50/72 (69.4)	18/35 (51.4)	8/13 (61.5)	0.191
High preoperative WBC level (>7200/μL)				
Tunneled CVC group (*n* = 12)	3/3 (100)	2/4 (50.0)	0/5 (0)	0.019
Non-tunneled CVC group (*n* = 114)	37/70 (52.9)	17/36 (47.2)	2/8 (25.0)	0.316
Both groups (*n* = 126)	40/73 (54.8)	19/40 (47.5)	2/13 (15.4)	0.032

CVC, central venous catheter; WBC, white blood cell.

## 4 Discussion

Although an AVF is widely regarded as the first-choice vascular access for hemodialysis, the rates of dialysis initiation with this vascular access modality remain suboptimal in many countries across the globe. The use of a CVC rather than an AVF to start dialysis therapy in ESRD patients may reflect the delay in referral to specialist nephrology/vascular surgery services, patients' poor overall health, patients’ financial constraints and physician and patient preferences. Among these, special attention should be paid to the use of a CVC as a bridge to AVF maturation because this practice can be avoided by improving the referral process and pre-ESRD patient education interventions.

Results from the Dialysis Outcomes and Practice Patterns Study (DOPPS) suggested that AVF survival was better in patients who did not have preexisting CVCs than in those who did [3]. Aside from AVF survival, the effects of preexisting CVCs on AVF maturation have been reported in a few studies [18,19,27]. The types of CVCs used in these reports varied from single to various types. Two previous studies that investigated the impact of the use of a single type of CVC showed conflicting results [19,27]. In a study conducted by Yoo et al., a significant association between prior tunneled CVC placement and AVF nonmaturation was noted [19]. In contrast, the other study conducted by Duque et al. did not observe a significant difference in AVF maturation between patients with prior tunneled CVC placement and those who had no history of preexisting CVC [27]. Focusing on the study investigating the effect of various types of CVCs used, a report by Rayner et al. on 3674 ESRD patients as part of the DOPPS found that the risk of AVF nonmaturation was increased by 1.8-fold among patients who had prior CVC placement, either tunneled or nontunneled, compared to those with no preexisting CVC [18].

The present study, which separately analyzed the impacts of the prior use of tunneled and nontunneled CVCs on AVF outcomes, also found a reduction in the odds of AVF maturation success among patients with a preexisting CVC. However, this effect was significant only in patients with preexisting nontunneled CVCs and not in those with preexisting tunneled CVCs. These results were in line with the finding of Duque et al. [27] in terms of the lack of an association between AVF nonmaturation and prior tunneled CVC placement and were consistent with the study of Rayner et al. [18] in terms of a relationship between AVF maturation failure and preexisting nontunneled CVC. A higher odds ratio for AVF maturation failure found in the present study compared to the study of Rayner et al. may be due to the difference in patient characteristics between the two studies. The odds ratio of 1.8 observed in the study of Rayner et al. was calculated from data of patients with a history of either tunneled or nontunneled CVC placement, whereas the odds ratio of 2.4 seen in the present study was the odds of AVF nonmaturation among patients who used only a nontunneled CVC.

The mechanisms by which preexisting CVCs affect AVF maturation remain elusive. One proposed explanation is that the CVC impedes the maturation of its ipsilateral AVF via mechanisms of hemodynamic changes as a consequence of catheter-induced central venous stenosis [28]. Nevertheless, the findings of one prior study did not corroborate this suggestion because it found no difference in AVF failure rates between patients with ipsilateral and contralateral CVC placements [28]. Apart from this hypothesis, systemic inflammation, a common condition occurring in the setting of CVC placement [23,24], has been proposed as a pathogenetic mechanism underlying neointimal hyperplasia [27], which is a foundation of AVF failure [[20], [21], [22]]. Goldstein et al. [24], who investigated the levels of inflammatory markers at the time of dialysis initiation and again 6 months later, found that patients with persistent CVC use from dialysis initiation through 6 months had consistently high inflammatory levels over the period, whereas the levels of inflammatory markers were attenuated in patients who changed from a catheter to an AVF. In the present study, patients with preexisting nontunneled CVC had significantly higher mean preoperative WBC and neutrophil counts than patients with no preexisting CVC, whereas the WBC profiles in patients with preexisting tunneled CVC and those with no preexisting dialysis catheter were not different. These results suggested that even without an infection, individuals who used a nontunneled CVC were more prone to inflammation than those using a tunneled catheter; therefore, persistent inflammation may be an explanation for the finding of a decrease in AVF maturation success among patients with preexisting nontunneled CVC but not among those with tunneled CVC that was observed in the present study.

A question may arise about the source of inflammation from the CVC in the absence of infection. One possible explanation is that the formation of biofilms might occur in some patients who have no clinical signs or symptoms of infection. Another likely reason is an immunological reaction against the CVC material itself [24]. Unfortunately, the effect of different CVC materials on inflammatory markers was not explored because it was beyond the scope of this study. Further research is needed to investigate this issue and search for the CVC material that causes the least inflammation.

One important finding of this study was an inverse relationship between the duration of CVC placement and AVF maturation success among patients whose preoperative WBC levels were above 7200/μL. This information should alert a clinician to the high possibility of AVF nonmaturation among patients with long-term CVC use who have a high preoperative WBC count. In this regard, patients who are at risk should receive comprehensive counseling regarding their probability of AVF maturation failure. Specialized treatment programs, including close monitoring after AVF creation or the use of arteriovenous grafts, should be applied in these patients.

Unlike previous studies that assessed only the effect of CVC use on AVF maturation success, this study was the first to explore its relation to the preoperative inflammatory status. The strength of this study was its large sample size. In addition, the effects of tunneled CVC and nontunneled CVC on AVF maturation success were separately evaluated to improve the precision of the results. The author was aware of the effect of surgical method on AVF maturation, so the same anastomosis technique was used for the AVF creation in the present study.

Nevertheless, this study was limited by being a retrospective study. Hence, some data might have been unavailable, such as blood flow measurements and the results of other inflammatory marker blood tests. C-reactive protein which may serve as a better indicator of systemic inflammation post CVC insertion [23,24] was not included in the practice guideline of the institution for preoperative care of patients undergoing AVF surgery. Although the antiplatelet or anticoagulation regimen was not uniformly prescribed to all patients, the author explored data but did not find a significant difference in rates of AVF maturation between patients who did not use antithrombotic drugs and those who did (67.5% *vs*. 64.3%, *p* = 0.438). However, future prospective research should control this factor by balancing the use of these agents. Another limitation was that the number of patients in the tunneled CVC group was limited, which might preclude drawing any definite conclusion. Finally, this study was conducted with a homogeneous cohort of ESRD patients from a single institution. Hence, the results might not be the same in other settings where people have different reference ranges for WBC counts or dissimilar material types of CVC are used.

## 5 Conclusion

This study demonstrated that preexisting nontunneled CVC but not tunneled CVC had a negative impact on AVF maturation success among patients undergoing first-time AVF creation. The evidence of this negative effect supported the recommendations of expert panels that nontunneled CVC should not be used for a long period. Given the high possibility of AVF maturation failure among patients with a history of long-term CVC use and preoperative WBC count above 7200/μL, future research is needed to evaluate whether applying pharmacologic interventions, such as preoperative anti-inflammatory medications, could improve AVF maturation in this group of patients.

### Provenance and peer review

Not commissioned, externally peer reviewed.

## Ethical approval

This study was approved by the Research Ethics Committee of the Faculty of Medicine Vajira Hospital, Navamindradhiraj University (No. 85/2561).

## Funding sources

This research was supported by the author’s institution under grant [#049/61].

## Please state any sources of funding for your research

This research received research grant from Faculty of Medicine Vajira Hospital, Navamindradhiraj University.

## Author contribution

The author designed study, obtained and interpreted data, wrote and approved the final manuscript.

## Registration of research studies

Thai Clinical Trials Registry. Number: TCTR20190624003.

Hyperlink to the registration: Clinical Trial Registry.

## Guarantor

Yuthapong Wongmahisorn.

## Consent

For this retrospective study, formal consent is not required.

## Declaration of competing interest

None.

## References

[1] Queeley G.L., Campbell E.F. (2018). Comparing treatment modalities for end-stage renal disease: a meta-analysis. Am. Health Drug Benefits..

[2] Santoro D., Benedetto F., Mondello P. (2014). Vascular access for hemodialysis: current perspectives. Int. J. Nephrol. Renovascular Dis..

[3] Pisoni R.L., Young E.W., Dykstra D.M. (2002). Vascular access use in Europe and the United States: results from the DOPPS. Kidney Int..

[4] Malas M.B., Canner J.K., Hicks C.W. (2015). Trends in incident hemodialysis access and mortality. JAMA Surg.

[5] Collins A.J., Foley R.N., Gilbertson D.T., Chen S.C. (2009). The state of chronic kidney disease, ESRD, and morbidity and mortality in the first year of dialysis. Clin. J. Am. Soc. Nephrol..

[6] Napalkov P., Felici D.M., Chu L.K., Jacobs J.R., Begelman S.M. (2013). Incidence of catheter-related complications in patients with central venous or hemodialysis catheters: a health care claims database analysis. BMC Cardiovasc. Disord..

[7] Almasri J., Alsawas M., Mainou M. (2016). Outcomes of vascular access for hemodialysis: a systematic review and meta-analysis. J. Vasc. Surg..

[8] Filiopoulos V., Hadjiyannakos D., Koutis I. (2011). Approaches to prolong the use of uncuffed hemodialysis catheters: results of a randomized trial. Am. J. Nephrol..

[9] Wang K., Wang P., Liang X., Lu X., Liu Z. (2015). Epidemiology of haemodialysis catheter complications: a survey of 865 dialysis patients from 14 haemodialysis centres in Henan province in China. BMJ Open.

[10] Zhang H., Li L., Jia H. (2017). Surveillance of dialysis events: one-year experience at 33 outpatient hemodialysis centers in China. Sci. Rep..

[11] The National Kidney Foundation Kidney Disease Outcomes Quality Initiative (2001). NKF-K/DOQI clinical practice guidelines for vascular access: update 2000. Am. J. Kidney Dis..

[12] Ponikvar R., Buturović-Ponikvar J. (2005). Temporary hemodialysis catheters as a long-term vascular access in chronic hemodialysis patients. Ther. Apher. Dial..

[13] Kovač J., Premru V., Buturović-Ponikvar J., Ponikvar R. (2011). Two single-lumen noncuffed catheters in the jugular vein as long-term vascular access: a preliminary report. Ther. Apher. Dial..

[14] Roca-Tey R., Arcos E., Comas J., Cao H., Tort J. (2016). Catalan Renal Registry Committee, Starting hemodialysis with catheter and mortality risk: persistent association in a competing risk analysis. J. Vasc. Access.

[15] Bonfante G.M., Gomes I.C., Andrade E.I., Lima E.M., Acurcio F.A., Cherchiglia M.L. (2011). Duration of temporary catheter use for hemodialysis: an observational, prospective evaluation of renal units in Brazil. BMC Nephrol..

[16] Wasse H., Speckman R.A., Frankenfield D.L., Rocco M.V., McClellan W.M. (2007). Predictors of delayed transition from central venous catheter use to permanent vascular access among ESRD patients. Am. J. Kidney Dis..

[17] Quinan P., Beder A., Berall M.J., Cuerden M., Nesrallah G., Mendelssohn D.C. (2011). A three-step approach to conversion of prevalent catheter-dependent hemodialysis patients to arteriovenous access. CANNT J..

[18] Rayner H.C., Pisoni R.L., Gillespie B.W. (2003). Creation, cannulation and survival of arteriovenous fistulae: data from the dialysis outcomes and practice Patterns study. Kidney Int..

[19] Yoo D.W., Yoon M., Jun H.J. (2014). Successful access rate and risk factor of vascular access surgery in arm for dialysis. Vasc. Specialist Int..

[20] Brahmbhatt A., Remuzzi A., Franzoni M., Misra S. (2016). The molecular mechanisms of hemodialysis vascular access failure. Kidney Int..

[21] Hu H., Patel S., Hanisch J.J. (2016). Future research directions to improve fistula maturation and reduce access failure. Semin. Vasc. Surg..

[22] Kaygin M.A., Halici U., Aydin A. (2013). The relationship between arteriovenous fistula success and inflammation, Ren. Fail.

[23] Dukkipati J.R., Molnar M.Z., Park J. (2014). Association of vascular access type with inflammatory marker levels in maintenance hemodialysis patients. Semin. Dial..

[24] Goldstein S.L., Ikizler T.A., Zappitelli M., Silverstein D.M., Ayus J.C. (2009). Non-infected hemodialysis catheters are associated with increased inflammation compared to arteriovenous fistulas. Kidney Int..

[25] Bashar K., Zafar A., Elsheikh S. (2015). Predictive parameters of arteriovenous fistula functional maturation in a population of patients with end-stage renal disease. PLoS One.

[26] Agha R.A., Borrelli M.R., Vella-Baldacchino M., Thavayogan R., Orgill D.P., STROCSS Group (2017). The STROCSS statement: strengthening the reporting of cohortstudies in surgery. Int. J. Surg..

[27] Duque J.C., Martinez L., Tabbara M. (2017). Arteriovenous fistula maturation in patients with permanent access created prior to or after hemodialysis initiation. J. Vasc. Access.

[28] Shingarev R., Barker-Finkel J., Allon M. (2012). Association of hemodialysis central venous catheter use with ipsilateral arteriovenous vascular access survival. Am. J. Kidney Dis..

